# Supplementary material for: miniMORPH: A Morphometry Pipeline for Low‐Field MRI in Infants
Source: Hum Brain Mapp. 2026 Jun 23;47(9):e70547. doi: 10.1002/hbm.70547 (PMC13288155; doi:10.1002/hbm.70547)
Supplement: Supplementary file 1 — Table S1: Software versions used in the miniMORPH pipeline. Table S2: ROI harmonisation between miniMORPH and SuperSynth. Table S3: Number of segmentations excluded following QC, stratified by template age and cohort. Segmentations were assessed on a region‐by‐region basis within each subject. Table S4: Heatmap representation of error metrics across regions and developmental stages. Heatmap of corrected mean squared error (MSE) and mean absolute error (MAE) between ULF miniMORPH‐derived volumes and HF reference segmentations, stratified by age (3–24 months) and region of interest. Colour intensity reflects the magnitude of the error, with darker shading indicating higher values. Sample sizes (N) for each age group are shown alongside the corresponding error metrics. Table S5: Supplementary Table 5. Table S6: Supplementary Table 6. Figure S1: Examples of QC outcomes and outlier handling. Cases with severe motion artefacts (top left), limited field‐of‐view resulting in cropped anatomy (top right), and reconstruction or acquisition artefacts (bottom left) were excluded due to clear segmentation failure or unreliable volumetric estimates. In contrast, cases with atypical but plausible anatomy, such as enlarged ventricles (bottom right), were retained following visual inspection, as segmentations were considered anatomically accurate. [file HBM-47-e70547-s001.docx]

**Supplementary Information**

***Supplementary Table 1.*** *Software versions used in the miniMORPH pipeline.*

| **Stage** | **Software** | **Version** | **Purpose** |
| --- | --- | --- | --- |
| Brain extraction | SynthStrip (FreeSurfer) | 7.4.1 | Skull stripping |
| Registration | ANTs | build 20220707 | Template construction and image registration |
| Atlas registration | SynthMorph (FreeSurfer implementation) | 7.4.1 | Atlas-to-template alignment |
| Segmentation | ANTs (AtroposN4) | build 20220707 | Tissue segmentation |
| Segmentation (benchmark) | SuperSynth (FreeSurfer) | 7.4.1 | Reference segmentation |
| Image processing | FSL | 6.0.7.17 | Masking, statistics, image operations |

***Supplementary Table 2.*** *ROI harmonisation between miniMORPH and SuperSynth.*

| **miniMORPH** | **SuperSynth** |
| --- | --- |
| supratentorial_tissue | WM-hypointensities, Left-Cerebral-White-Matter, Left-Cerebral-Cortex, Left-AmygdalaLeft-Hippocampus, Left-Accumbens-area, Left-HypoThal-noMB, Left-FornixLeft-MammillaryBody, Left-Basal-Forebrain, Left-SeptalNuc, Right-Cerebral-White-MatterRight-Cerebral-Cortex, Right-Hippocampus, Right-Amygdala, Right-Accumbens-areaRight-HypoThal-noMB, Right-Fornix, Right-MammillaryBody, Right-Basal-ForebrainRight-SeptalNuc, Optic-Chiasm, Left-Thalamus, Left-CaudateLeft-Putamen, Left-Pallidum, Right-Thalamus, Right-CaudateRight-Putamen, Right-Pallidum |
| ventricles | 3rd-Ventricle, 4th-Ventricle, Left-Lateral-Ventricle, Left-Inf-Lat-VentRight-Lateral-Ventricle, Right-Inf-Lat-Vent |
| cerebellum | Left-Cerebellum-White-Matter, Left-Cerebellum-Cortex, Right-Cerebellum-White-Matter, Right-Cerebellum-Cortex |
| left_thalamus | Left-Thalamus |
| left_caudate | Left-Caudate |
| left_putamen | Left-Putamen |
| left_globus_pallidus | Left-Pallidum |
| right_thalamus | Right-Thalamus |
| right_caudate | Right-Caudate |
| right_putamen | Right-Putamen |
| right_globus_pallidus | Right-Pallidum |
| icv | CSF, Left-Cerebellum-White-Matter, Left-Cerebellum-Cortex, Right-Cerebellum-White-MatterRight-Cerebellum-Cortex, WM-hypointensities, Left-Cerebral-White-Matter, Left-Cerebral-CortexLeft-Amygdala, Left-Hippocampus, Left-Accumbens-area, Left-HypoThal-noMBLeft-Fornix, Left-MammillaryBody, Left-Basal-Forebrain, Left-SeptalNucRight-Cerebral-White-Matter, Right-Cerebral-Cortex, Right-Hippocampus, Right-AmygdalaRight-Accumbens-area, Right-HypoThal-noMB, Right-Fornix, Right-MammillaryBodyRight-Basal-Forebrain, Right-SeptalNuc, Optic-Chiasm, Left-ThalamusLeft-Caudate, Left-Putamen, Left-Pallidum, Right-ThalamusRight-Caudate, Right-Putamen, Right-Pallidum |

***Supplementary Table 3.*** *Number of segmentations excluded following QC, stratified by template age and cohort. Segmentations were assessed on a region-by-region basis within each subject.*

| **Region** | **Template Age** | | | | |
| --- | --- | --- | --- | --- | --- |
| **UCT Khula** | | | | | |
|  | **3M** | **6M** | **12M** | **18M** | **24M** |
| Supratentorial Tissue | 2 | 3 | 2 | 0 | 0 |
| Supratentorial CSF | 2 | 1 | 2 | 0 | 0 |
| Ventricles | 2 | 2 | 1 | 0 | 0 |
| Cerebellum | 2 | 2 | 1 | 0 | 1 |
| Cerebellum CSF | 2 | 1 | 1 | 0 | 0 |
| Brainstem | 2 | 2 | 2 | 0 | 0 |
| Brainstem CSF | 2 | 4 | 2 | 1 | 0 |
| Left Thalamus | 2 | 2 | 1 | 0 | 0 |
| Left Caudate | 2 | 3 | 1 | 0 | 0 |
| Left Putamen | 2 | 2 | 2 | 0 | 0 |
| Left Globus Pallidus | 2 | 2 | 2 | 0 | 0 |
| Right Thalamus | 2 | 2 | 2 | 0 | 0 |
| Right Caudate | 2 | 2 | 3 | 0 | 0 |
| Right Putamen | 2 | 2 | 2 | 0 | 0 |
| Right Globus Pallidus | 2 | 2 | 2 | 0 | 0 |
| Posterior Callosum | 2 | 1 | 2 | 0 | 0 |
| Mid Posterior Callosum | 3 | 4 | 2 | 0 | 0 |
| Central Callosum | 3 | 4 | 3 | 0 | 0 |
| Mid Anterior Callosum | 2 | 3 | 2 | 0 | 0 |
| Anterior Callosum | 3 | 3 | 3 | 0 | 0 |
| ICV | 2 | 3 | 2 | 0 | 0 |
| **Uganda PRIMES** | | | | | |
|  | **3M** | **6M** | **12M** | **18M** | **24M** |
| Supratentorial Tissue | 2 | 16 | 11 | - | - |
| Supratentorial CSF | 2 | 16 | 11 | - | - |
| Ventricles | 2 | 16 | 12 | - | - |
| Cerebellum | 2 | 16 | 11 | - | - |
| Cerebellum CSF | 2 | 16 | 11 | - | - |
| Brainstem | 2 | 16 | 11 | - | - |
| Brainstem CSF | 2 | 16 | 11 | - | - |
| Left Thalamus | 2 | 17 | 11 | - | - |
| Left Caudate | 3 | 16 | 11 | - | - |
| Left Putamen | 2 | 16 | 11 | - | - |
| Left Globus Pallidus | 2 | 16 | 11 | - | - |
| Right Thalamus | 2 | 17 | 11 | - | - |
| Right Caudate | 3 | 17 | 11 | - | - |
| Right Putamen | 2 | 16 | 11 | - | - |
| Right Globus Pallidus | 2 | 16 | 11 | - | - |
| Posterior Callosum | 2 | 16 | 11 | - | - |
| Mid Posterior Callosum | 2 | 16 | 11 | - | - |
| Central Callosum | 2 | 16 | 11 | - | - |
| Mid Anterior Callosum | 3 | 17 | 12 | - | - |
| Anterior Callosum | 3 | 17 | 12 | - | - |
| ICV | 2 | 16 | 11 | - | - |

***Supplementary Table 4.*** *Heatmap representation of error metrics across regions and developmental stages. Heatmap of corrected mean squared error (MSE) and mean absolute error (MAE) between ULF miniMORPH-derived volumes and HF reference segmentations, stratified by age (3–24 months) and region of interest. Colour intensity reflects the magnitude of the error, with darker shading indicating higher values. Sample sizes (N) for each age group are shown alongside the corresponding error metrics.*


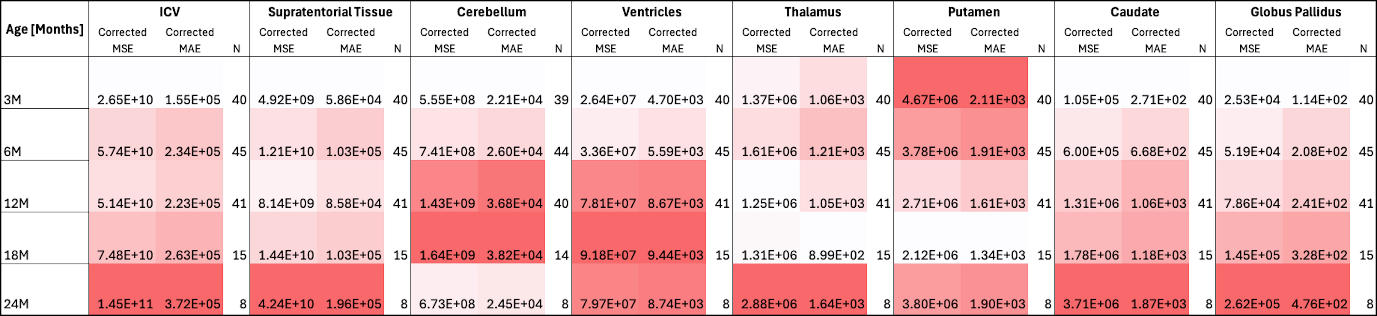


***Supplementary Table 5.***

| **Uncorrected** | | | | | | |
| --- | --- | --- | --- | --- | --- | --- |
| **Region** | **Sex Effect** | **Corrected p**  **(Sex)** | **Age Effect** | **Corrected p (Age)** | **Quadratic Age Effect**  **(p-value)** | **Corrected p (Age^2^)** |
| **Supratentorial tissue** | 63183.9614 | **2.68E-26** | 39117.4968 | **7.12E-96** | -892.7499 | **3.40E-30** |
| **Supratentorial CSF** | 10214.0069 | **1.26E-06** | 8791.06053 | **5.63E-45** | -259.3807 | **1.20E-23** |
| **Ventricles** | 2366.81877 | **3.96E-08** | 1206.84906 | **3.48E-20** | -31.296255 | **2.71E-08** |
| **Cerebellum** | 7053.71233 | **4.26E-16** | 7231.26777 | **1.88E-163** | -197.21719 | **2.91E-73** |
| **Cerebellum CSF** | 1309.15432 | **1.46E-06** | 1692.70515 | **4.77E-103** | -45.808409 | **1.61E-45** |
| **Brainstem** | 782.941131 | **1.50E-15** | 496.848946 | **9.70E-60** | -11.079133 | **3.98E-18** |
| **Brainstem CSF** | 1472.71826 | **3.09E-21** | 769.7384 | **1.99E-56** | -18.192016 | **3.52E-19** |
| **Thalamus** | 728.780705 | **1.47E-19** | 107.058132 | **2.16E-60** |  | 1 |
| **Caudate** | 282.325646 | **6.08E-11** | 100.165797 | **2.50E-13** | -1.3969815 | 0.13967485 |
| **Putamen** | 520.590705 | **2.52E-14** | 371.001492 | **2.53E-69** | -7.6235112 | **6.79E-18** |
| **Globus pallidus** | 130.705346 | **6.51E-09** | 91.1647054 | **4.61E-41** | -2.5102742 | **6.76E-19** |
| **Posterior callosum** | 589.524984 | **1.47E-19** | 364.310847 | **1.53E-68** | -8.2974108 | **1.21E-21** |
| **Mid-posterior callosum** | 76.7037258 | **6.56E-12** | 57.3489501 | **3.81E-63** | -1.1905914 | **1.25E-16** |
| **Central callosum** | 117.452057 | **1.72E-11** | 68.3637674 | **2.13E-37** | -1.4867587 | **7.99E-11** |
| **Mid-anterior callosum** | 182.547097 | **3.02E-11** | 49.9692895 | **2.83E-08** | -1.0168306 | **0.04515241** |
| **Anterior callosum** | 493.51014 | **5.73E-14** | 182.569712 | **1.12E-18** | -3.2440844 | **0.00115071** |
| **ICV-corrected** | | | | | | |
| **Supratentorial tissue** | 0.00443259 | **0.01142901** | -0.0005605 | 1 | 6.70E-05 | **0.00151859** |
| **Supratentorial CSF** | -0.0053064 | **1.35E-05** | -0.0034477 | **3.66E-22** | 5.97E-05 | **0.00039885** |
| **Ventricles** | 0.00076109 | 0.3123177 | 1.39E-05 | 1 |  | 1 |
| **Cerebellum** | 0.00064017 | 1 | 0.00364034 | **9.26E-90** | -0.000115 | **6.23E-54** |
| **Cerebellum CSF** | -0.0006557 | 0.055598 | 0.00033732 | **3.90E-05** | -1.27E-05 | **0.0002275** |
| **Brainstem** | -1.84E-05 | 1 | 7.64E-06 | 1 |  | 1 |
| **Brainstem CSF** | 0.00047016 | **0.00062178** | 6.18E-05 | **8.47E-10** |  | 1 |
| **Thalamus** | -0.0001369 | 0.19132756 | -0.0005496 | **3.19E-215** | 1.64E-05 | **2.37E-115** |
| **Caudate** | -7.34E-05 | 0.13909017 | -0.0001628 | **1.91E-72** | 5.18E-06 | **3.40E-44** |
| **Putamen** | -2.48E-05 | 1 | 3.22E-05 | **8.96E-24** |  | 1 |
| **Globus pallidus** | -5.50E-05 | **0.00408543** | -4.97E-05 | **7.32E-24** | 1.11E-06 | **2.53E-07** |
| **Posterior callosum** | 6.15E-05 | 1 | 1.30E-05 | **0.001245** |  | 1 |
| **Mid-posterior callosum** | -7.32E-06 | 1 | 4.03E-06 | **3.13E-09** |  | 1 |
| **Central callosum** | 1.61E-05 | 1 | 2.24E-06 | 0.5562203 |  | 1 |
| **Mid-anterior callosum** | 7.64E-06 | 1 | -8.91E-05 | **4.26E-42** | 2.55E-06 | **9.94E-21** |
| **Anterior callosum** | 3.60E-05 | 1 | -0.0001639 | **4.19E-29** | 5.29E-06 | **2.14E-18** |

***Supplementary Table 6.***

| **Uncorrected** | | | | | | | | |
| --- | --- | --- | --- | --- | --- | --- | --- | --- |
| **Region** | **Group Effect** | **Corrected p (Group)** | **Age Effect** | **Corrected p (Age)** | **Age x Group Interaction Effect** | **Corrected p (Age x Group Interaction)** | **Sex Effect** | **Corrected p (Sex)** |
| **Supratentorial tissue** | 1.25E+04 | 1.00E+00 | 1.49E+04 | **1.49E-100** | 9.65E+03 | **1.40E-51** | 4.91E+04 | **4.91E-18** |
| **Supratentorial CSF** | 1.87E+04 | **4.30E-17** | 1.77E+03 | **7.77E-04** | 2.98E+03 | **2.03E-06** | 9.38E+03 | **5.13E-07** |
| **Ventricles** | 1.33E+03 | 5.30E-02 | 2.98E+02 | 9.09E-02 | 4.66E+02 | **8.06E-04** | 2.09E+03 | **2.31E-09** |
| **Cerebellum** | 5.57E+03 | **2.95E-08** | 2.46E+03 | **6.19E-123** | 1.71E+03 | **5.47E-21** | 5.65E+03 | **1.46E-13** |
| **Cerebellum CSF** | 6.88E+02 | 9.18E-02 | 5.33E+02 | **6.70E-17** | 5.07E+02 | **1.94E-16** | 1.26E+03 | **2.85E-10** |
| **Brainstem** | 2.29E+01 | 1.00E+00 | 1.88E+02 | **1.02E-25** | 1.40E+02 | **5.72E-19** | 5.95E+02 | **2.74E-10** |
| **Brainstem CSF** | 1.14E+03 | **3.42E-10** | 1.89E+02 | **1.29E-06** | 3.06E+02 | **1.35E-10** | 1.27E+03 | **1.74E-27** |
| **Thalamus** | 6.50E+01 | 1.00E+00 | 5.26E+01 | **3.31E-16** | 6.26E+01 | **7.97E-21** | 6.00E+02 | **1.75E-14** |
| **Caudate** | 2.44E+02 | **1.09E-05** | 4.58E+01 | **4.48E-11** | 2.92E+01 | **5.43E-12** | 1.75E+02 | **1.47E-04** |
| **Putamen** | 3.48E+02 | **2.61E-05** | 1.36E+02 | **3.39E-29** | 1.08E+02 | **1.47E-08** | 3.96E+02 | **1.71E-10** |
| **Globus pallidus** | 1.01E+02 | **2.09E-04** | 3.43E+01 | **1.26E-07** | 1.93E+01 | 5.85E-02 | 1.06E+02 | **8.91E-07** |
| **Posterior callosum** | 1.40E+02 | 1.00E+00 | 1.35E+02 | **1.64E-14** | 9.93E+01 | **1.31E-10** | 4.51E+02 | **6.48E-14** |
| **Mid-posterior callosum** | -1.88E+00 | 1.00E+00 | 2.25E+01 | **1.62E-13** | 1.68E+01 | **8.56E-06** | 5.77E+01 | **1.59E-07** |
| **Central callosum** | 4.12E+00 | 1.00E+00 | 2.78E+01 | **2.04E-24** | 1.75E+01 | **3.57E-23** | 9.51E+01 | **3.33E-08** |
| **Mid-anterior callosum** | -3.23E+01 | 1.00E+00 | 1.31E+01 | **3.96E-04** | 1.84E+01 | **1.55E-02** | 1.51E+02 | **1.21E-07** |
| **Anterior callosum** | -1.29E+02 | 1.00E+00 | 5.25E+01 | **9.10E-03** | 7.20E+01 | **6.90E-03** | 4.09E+02 | **3.81E-09** |
| **ICV-corrected** | | | | | | | | |
| **Supratentorial tissue** | -1.40E-02 | **2.82E-26** | 8.93E-04 | 1.38E-01 | -5.63E-04 | 1.00E+00 | 2.55E-03 | **3.85E-02** |
| **Supratentorial CSF** | 1.25E-02 | **1.43E-40** | -2.08E-03 | **1.87E-12** | -3.43E-04 | 1.00E+00 | -4.00E-03 | **2.40E-05** |
| **Ventricles** | 5.33E-04 | 1.00E+00 | -7.24E-05 | 1.00E+00 | 1.76E-04 | 1.00E+00 | 8.02E-04 | 1.22E-01 |
| **Cerebellum** | 2.32E-03 | **7.24E-03** | 1.19E-03 | **6.18E-14** | 6.70E-04 | **1.21E-03** | 9.37E-04 | **4.65E-02** |
| **Cerebellum CSF** | -2.90E-04 | 1.00E+00 | 3.58E-05 | 1.00E+00 | 1.56E-04 | 3.50E-01 | -3.32E-04 | 3.71E-01 |
| **Brainstem** | -3.37E-04 | **1.28E-11** | -4.63E-06 | 1.00E+00 | 3.32E-06 | 1.00E+00 | -3.87E-05 | 1.00E+00 |
| **Brainstem CSF** | 6.87E-04 | **3.68E-17** | -2.14E-05 | 1.00E+00 | 1.20E-04 | **1.70E-02** | 5.17E-04 | **1.41E-05** |
| **Thalamus** | -2.30E-04 | **2.29E-10** | -1.81E-04 | **9.35E-21** | -1.10E-04 | **2.89E-06** | -1.23E-04 | 9.72E-01 |
| **Caudate** | -3.87E-04 | **8.89E-24** | -5.13E-05 | **3.45E-07** | -3.12E-05 | **3.52E-02** | -1.28E-04 | **6.53E-11** |
| **Putamen** | 1.07E-04 | 3.19E-01 | 1.60E-05 | 9.21E-01 | 8.50E-06 | 1.00E+00 | -2.85E-05 | 1.00E+00 |
| **Globus pallidus** | 2.93E-05 | 1.00E+00 | -1.19E-05 | 1.97E-01 | -1.79E-05 | **1.06E-02** | -4.93E-05 | **8.18E-05** |
| **Posterior callosum** | -1.11E-04 | 2.92E-01 | 7.16E-06 | 1.00E+00 | 6.67E-06 | 1.00E+00 | 2.74E-05 | 1.00E+00 |
| **Mid-posterior callosum** | -4.56E-05 | **8.16E-09** | 1.47E-06 | 1.00E+00 | 2.46E-06 | 1.00E+00 | -1.08E-05 | 1.00E+00 |
| **Central callosum** | -4.73E-05 | 9.11E-02 | 2.22E-06 | 1.00E+00 | -7.30E-07 | 1.00E+00 | 1.10E-05 | 1.00E+00 |
| **Mid-anterior callosum** | -9.92E-05 | **1.63E-05** | -3.51E-05 | **9.91E-07** | -1.54E-05 | 6.06E-01 | -2.53E-06 | 1.00E+00 |
| **Anterior callosum** | -3.16E-04 | **3.99E-06** | -7.28E-05 | **2.98E-05** | -1.29E-05 | 1.00E+00 | 1.82E-05 | 1.00E+00 |


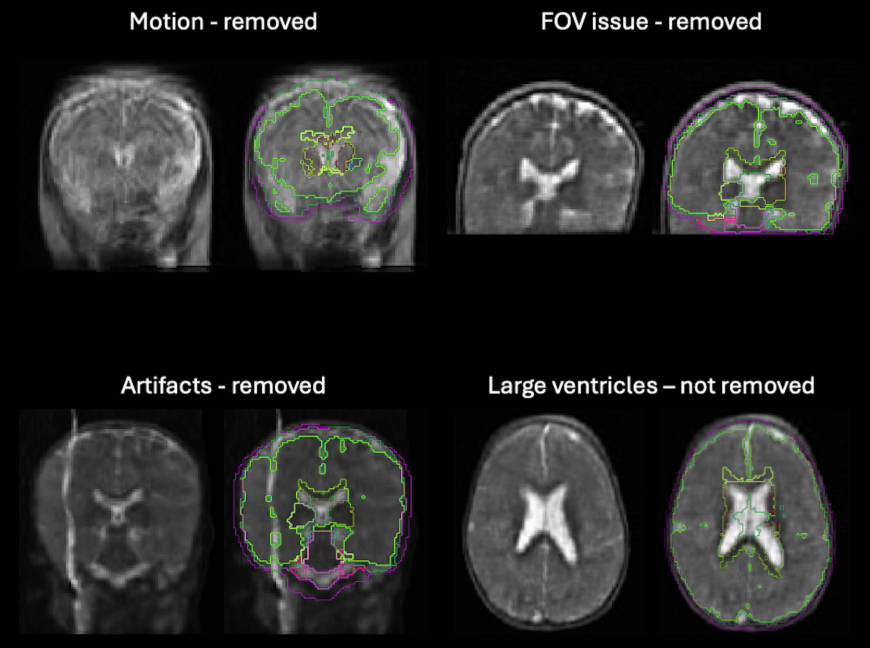
***Supplementary Figure 1. Examples of QC outcomes and outlier handling.*** *Cases with severe motion artefacts (top left), limited field-of-view resulting in cropped anatomy (top right), and reconstruction or acquisition artefacts (bottom left) were excluded due to clear segmentation failure or unreliable volumetric estimates. In contrast, cases with atypical but plausible anatomy, such as enlarged ventricles (bottom right), were retained following visual inspection, as segmentations were considered anatomically accurate.*
